# Pregnancy-specific stress and sensitive caregiving during the transition to motherhood in adolescents

**DOI:** 10.1186/s12884-021-03903-5

**Published:** 2021-06-29

**Authors:** Pamela Scorza, Emily C. Merz, Marisa Spann, Emily Steinberg, Tianshu Feng, Seonjoo Lee, Elizabeth Werner, Bradley S. Peterson, Catherine Monk

**Affiliations:** 1 Department of Psychiatry, Columbia University Medical Center, New York, NY USA; 2 New York State Psychiatric Institute, New York, NY USA; 3 Department of Psychology, Colorado State University, 0000- 0003-1950-2345 Fort Collins, CO USA; 4 Department of Psychology, Fordham University, New York, NY USA; 5 Department of Pediatrics, Children’s Hospital Los Angeles, University of Southern California, Los Angeles, CA USA; 6 Division of Child and Adolescent Psychiatry, Keck School of Medicine, University of Southern California, Los Angeles, CA USA; 7 Institute for the Developing Mind, Children’s Hospital Los Angeles, Los Angeles, CA USA; 8 Department of Obstetrics and Gynecology, Columbia University Medical Center, New York, NY USA

**Keywords:** Pregnancy, Adolescents, Maternal caregiving, Childhood trauma, Pregnancy-specific Stress

## Abstract

**Background:**

Maternal prenatal stress is associated with worse socio-emotional outcomes in offspring throughout childhood. However, the association between prenatal stress and later caregiving sensitivity is not well understood, despite the significant role that caregiving quality plays in child socio-emotional development. The goal of this study was to examine whether dimensions of pregnancy-specific stress are correlated with observer-based postnatal maternal caregiving sensitivity in pregnant adolescents.

**Methods:**

Healthy, nulliparous pregnant adolescents *(n* = 244; 90 % LatinX) reported on their pregnancy-specific stress using the Revised Prenatal Distress Questionnaire (NuPDQ). Of these 244, 71 participated in a follow-up visit at 14 months postpartum. Videotaped observations of mother-child free play interactions at 14 months postpartum were coded for maternal warmth and contingent responsiveness. Confirmatory factor analysis of the NuPDQ supported a three-factor model of pregnancy-specific stress, with factors including stress about the social and economic context, baby’s health, and physical symptoms of pregnancy.

**Results:**

Greater pregnancy-specific stress about social and economic context and physical symptoms of pregnancy was associated with reduced maternal warmth but not contingent responsiveness.

**Conclusions:**

Heightened maternal stress about the social and economic context of the perinatal period and physical symptoms of pregnancy may already signal future difficulties in caregiving and provide an optimal opening for early parenting interventions.

## Background

Research on Prenatal Programming, or the Developmental Origins of Health and Disease, suggests that maternal stress during the prenatal period serves as a signal to which the developing organism adapts and which impacts the trajectory of fetal development. A recent meta-analysis of 71 studies found a weighted average effect size of 1.66 (95 % CI = 1.54–1.79) for the association between prenatal maternal stress and offspring socioemotional outcomes up to age 18 [1]. A body of evidence also suggests that caregiving sensitivity has an important effect on offspring socio-emotional outcomes [2–6]. However, studies that examine the association of prenatal stress with offspring outcomes years later often do not consider caregiving sensitivity as a potential mediator or moderator of the relationship between prenatal stress and offspring socio-emotional development, even though and prenatal maternal stress has been demonstrated to be associated with suboptimal caregiving sensitivity [7–10]. Caregiving sensitivity encompasses two core dimensions of warmth and contingent responsiveness. Maternal warmth refers to physical and verbal affection expressed toward the child, as well as acceptance of the child’s needs and interests [11]. Contingent responsiveness refers to prompt, appropriate behaviors in response to the child’s cues, such as following the child’s lead and pacing and displaying flexibility in adjusting to the child’s play interests [12].

The current study addresses several gaps in this research: (1) *“Stress” rather than depression and anxiety*: The majority of existing studies focus on the effects of maternal depression and anxiety on parenting, and it is not clear whether prenatal stress, rather than the narrower category of depression and anxiety symptoms, influences caregiving sensitivity. In rodents, prenatal stress reduces nurturing maternal behavior [13] but this has not been explicitly assessed in humans. (2) *What kind of stress matters?* Measuring general stress during pregnancy, without assessing stress that women experience because of pregnancy itself, may result in underestimates of the magnitude of stress that pregnant women experience [14, 15]. Pregnancy-specific stress has recently been examined in relation to outcomes for the mother-infant dyad [16]. Pregnancy-specific stress (sometimes termed “pregnancy-related anxiety” or “pregnancy-related distress”) presents as a separate clinical phenomenon distinct from measures of general stress or anxiety [16, 17] and has shown stronger associations with neuroendocrine changes during pregnancy, birth outcomes, and postnatal mood compared to general stress during pregnancy [16, 18]. Still, it is unclear whether specific dimensions of pregnancy-specific stress are associated with maternal/child outcomes. Identifying aspects of maternal pregnancy-specific stress that most impact caregiving sensitivity could inform the design of more effective interventions for at-risk mother-child dyads, ultimately improving the health and development of these vulnerable mothers and children [19–21]. (3) *Stress and caregiving in a high-risk population.* We examine stress and caregiving sensitivity in a particularly at-risk group— pregnant adolescents, the majority of whom were also LatinX minority adolescents in our sample. Compared with children of adult mothers, children of adolescent mothers are at higher risk for adverse development and socio-emotional problems, [22–27], and a recent study found that LatinX women reported more pregnancy stress than non-LatinX white women [28].

### Current Study

The goal of this study was to examine the associations of pregnancy-specific stress with postnatal caregiving sensitivity in pregnant adolescents. We hypothesized that higher pregnancy-specific stress would be associated with lower maternal caregiving sensitivity. Understanding these pathways in a high-risk population could inform prevention strategies to promote healthy child developmental trajectories.

## Methods

### Participants

*Recruitment*. Participants are from a prospective longitudinal study of pregnant adolescents recruited between 2009 and 2012 through the Departments of Obstetrics and Gynecology at Columbia University Irving Medical Center and Weill Cornell Medical College, and flyers posted in the Columbia University Irving Medical Center vicinity.

Pregnant women ages 14–19 receiving prenatal care and not experiencing significant pregnancy complications were recruited in the original study, which sought to assess the influence of maternal prenatal stress and poor nutrition on offspring cognitive development. Participants were excluded if they lacked fluency in English, were multiparous, had major pregnancy complications (mild complications such as a yeast infection or urinary tract infection were permitted), smoked tobacco, or used recreational drugs, nitrates, steroids, systemic migraine medications, stimulants, major and minor tranquilizers, or psychiatric medications. Random urine drug screens were conducted during pregnancy. On random urine toxicology screens, one participant tested positive for cannabinoids during pregnancy and was excluded. One pregnancy ended in fetal death; this participant was excluded from analysis. The original study sample size, after these exclusions was *n* = 244. We report a secondary analysis using a subsample of 71 who returned for a 14-month postnatal visit and had complete information on pregnancy-specific stress.

Participants completed questionnaires during lab visits at early (13–16 weeks), middle (24–27 weeks), and late (34–37 weeks) pregnancy. They returned to the lab with their infants at 14 months postpartum. During this lab visit, they completed a 10-minute mother-child free play session, which was videotaped for later coding. All participants provided written informed consent, and all procedures were approved by the Institutional Review Board of the New York State Psychiatric Institute/Columbia University Irving Medical Center.

### Measures

Pregnancy-specific stress was measured using the Revised Prenatal Distress Questionnaire (NuPDQ) [29, 30], which focuses on specific worries and concerns related to pregnancy. It asks how much women feel “bothered, upset, or worried” during pregnancy by given items, which are rated on a 3-point scale ranging from 0 (*not at all)* to 2 (*very much)*. Items include “about whether you might have an unhealthy baby,” “about working or caring for your family during your pregnancy,” “about changes in your relationships with other people due to having a baby”. There are 9, 12, and 17 items in the first-, second-, and third-trimester versions of the NuPDQ, respectively [30]. The NuPDQ has demonstrated reliability and convergent, concurrent, and predictive validity in other samples [14], and in this study, internal consistency for the NuPDQ was acceptable (Cronbach’s α = 0.76). NuPDQ items were administered at the second and third lab visit during pregnancy. Responses during the second lab visit were used in this study because of the clinical utility of identifying stress earlier in pregnancy, allowing more time for intervention.

*Maternal warmth and contingent responsiveness* were coded from videotapes of the mother-child free play sessions at 14 months. Global ratings of warmth and contingent responsiveness were made using 5-point scales, with 1 = *almost never*, 2 = *some of the time*, 3 = *half the time*, 4 = *most of the time*, and 5 = *almost always*. These rating scales were adapted from well-validated rating scales that have been used extensively in previous work (e.g., [31, 32]. Although this coding scheme (31) contains other rating scales, the warmth and contingent responsiveness scales were the only ones used for this study. This approach was taken because the warmth and contingent responsiveness scales were most relevant to the construct of maternal sensitivity [32].

Maternal warmth refers to physical and verbal affection expressed toward the child, as well as acceptance of the child’s needs and interests [11]. Ratings of warmth were based on the following developmentally-appropriate indicators: engagement with the child, expressions of positive affect toward the child (e.g., smiling, supportive tone of voice), praise, encouragement, and physical affection. Mothers who were rated as high in warmth frequently displayed the above behaviors and did not display any negativity (e.g., anger, criticism) toward their children. Contingent responsiveness refers to prompt, appropriate behaviors in response to the child’s cues, such as following the child’s lead and pacing and displaying flexibility in adjusting to the child’s interests [12, 33–35]. Ratings of contingent responsiveness were based on the following indicators: prompt and appropriate responses to child signals; recognizing, engaging with, and facilitating child’s play interests; and pacing that is in sync with the child. Mothers who were rated as high in contingent responsiveness frequently displayed the above behaviors and did not display any controlling or intrusive behavior toward their children (e.g., controlling which toys the child played with).

A master coder and a trainee coder completed the ratings of maternal warmth and contingent responsiveness. The trainee coder was provided with a manual and spent three weeks in training to achieve reliability and was supervised during coding to monitor drift and reliability. Inter-rater reliability was adequate, as intraclass correlation coefficients computed for 20 % of the sample were 0.66 and 0.75 for warmth and contingent responsiveness, respectively. Coders were not involved in data collection for this study and were not aware of the prenatal or other maternal and infant characteristics of the sample during coding.

#### Maternal perceived stress

Maternal stress at the 14-month postnatal timepoint was considered as a covariate. This was measured by the Perceived Stress Scale, [36] a 14-item instrument designed to measure the degree to which situations in one’s life over the last month are appraised as stressful. Items include “unable to control important things in life,” “confident about ability to handle personal problems,” and “difficulties were piling up so high that you could not overcomes them. A 5-point likert scale provides response options from “never” to “very often.” Responses were summed across items to create a total score. The PSS has previously been used effectively in pregnant adolescents [37].

### Analytic Approach

We used Mplus (version 6.0) to run a 3-factor confirmatory factor analysis (CFA) model based on previous work, with the three factors containing items related to (1) Concerns about the baby’s health (2) Concerns about physical symptoms during pregnancy and (3) Concerns about the social and economic context related to having a baby [38]. Because NuPDQ item responses were categorical variables, the factor analysis was based on polychoric correlations using robust weighted least squares estimators. The weighted least squares estimator does not assume normally distributed variables and provides the best option for modeling categorical or ordered data [39]. With a sample size of 244, the dataset exceeded minimum sample size guidelines for CFA [40]. Goodness-of-fit was measured by the Root Mean Square Error of Approximation (RMSEA, recommended to be 0.06 or below), and the Comparative Fit Index (CFI), which is recommended to be close to 0.95 or above, [41] although these guidelines are highly dependent on model estimators and parameters, and they may be too conservative, particularly in cases with many indicators and several factors [42], as in the case in this analysis. Full information maximum likelihood estimation was used to handle missing data.

Using SAS software (version 9.4), ordinal logistic regression was employed to examine associations between the NuPDQ factors and maternal warmth and contingent responsiveness, with *p* < .05 to denote significance. Ordinal values of observationally-coded maternal warmth and contingent responsiveness were as follows: *almost never, some of the time, half the time, most of the time, almost always*. Ordinal logistic regression can be used to estimate associations between an ordinal dependent variable and a set of independent variables [43]. Control variables included maternal age and infant sex, given some evidence of differences in caregiving sensitivity by child sex [44]. Maternal stress at the 14-month postnatal time period (PSS) was also considered as a covariate. This was measured by the Perceived Stress Scale, [36] a 14-item instrument designed to measure the degree to which situations in one’s life over the last month are appraised as stressful. A 5-point likert scale provides response options from “never” to “very often.” Responses were summed across items to create a total score. Prenatal depression and anxiety symptoms were not included as covariates because of the conceptual overlap and high co-linearity between depression/anxiety symptoms and pregnancy-specific stress (r = .36 for depression symptoms, measured by the Reynolds Adolescent Depression Scale and r = .63 for anxiety symptoms, measured by the Perceived Stress Scale) For this analysis, we were more interested in the construct of pregnancy-specific stress.

## Results

### Participant characteristics

Participants were 71 nulliparous pregnant adolescents, ages 14–19 years and between 13 and 27 gestational weeks. All adolescents had a healthy pregnancy at the time of recruitment. Sample characteristics are provided in Table 1.

**Table 1:** Descriptive statistics for sample characteristics (*N* = 71)

	*M*	*SD*
Maternal age (years)	17.56	1.35
Maternal education (years)^a^	11.06	1.13
Child gestational age at birth (weeks)	39.34	1.31
Child birth weight (grams)^a^	3195.76	435.71
	** %**	***n***
Maternal race/ethnicity		
Hispanic/Latina	85 %	60
African American	15 %	11
Child sex (female)	42 %	30

^a^*n* = 68

Of the 244 adolescents providing NuPDQ data during the second study visit of pregnancy, 71 returned to the lab with their infants for the 14-month postnatal research visit. These 71 dyads participated in a free play session, which was used to code maternal sensitivity. Taken together, 71 mother-infant dyads had both NuPDQ and maternal sensitivity data. Participants with 14-month caregiving sensitivity data compared to those who did not return for the 14-month visit did not differ significantly in terms of NuPDQ total score, *t*(242) = − 0.17, *p* = .86, maternal education, *t*(230) = − 0.27, *p* = .78, maternal race/ethnicity, χ^2^(1) = 3.15, *p* = .08, baby gestational age at birth, *t*(229) = − 0.96, *p* = .34, baby birth weight, *t*(220) = 0.28, *p* = .78, or baby sex, χ^2^(1) = 0.02, *p* = .89.

### Confirmatory Factor Analysis of Pregnancy-Related Stress

**Fig. 1:**
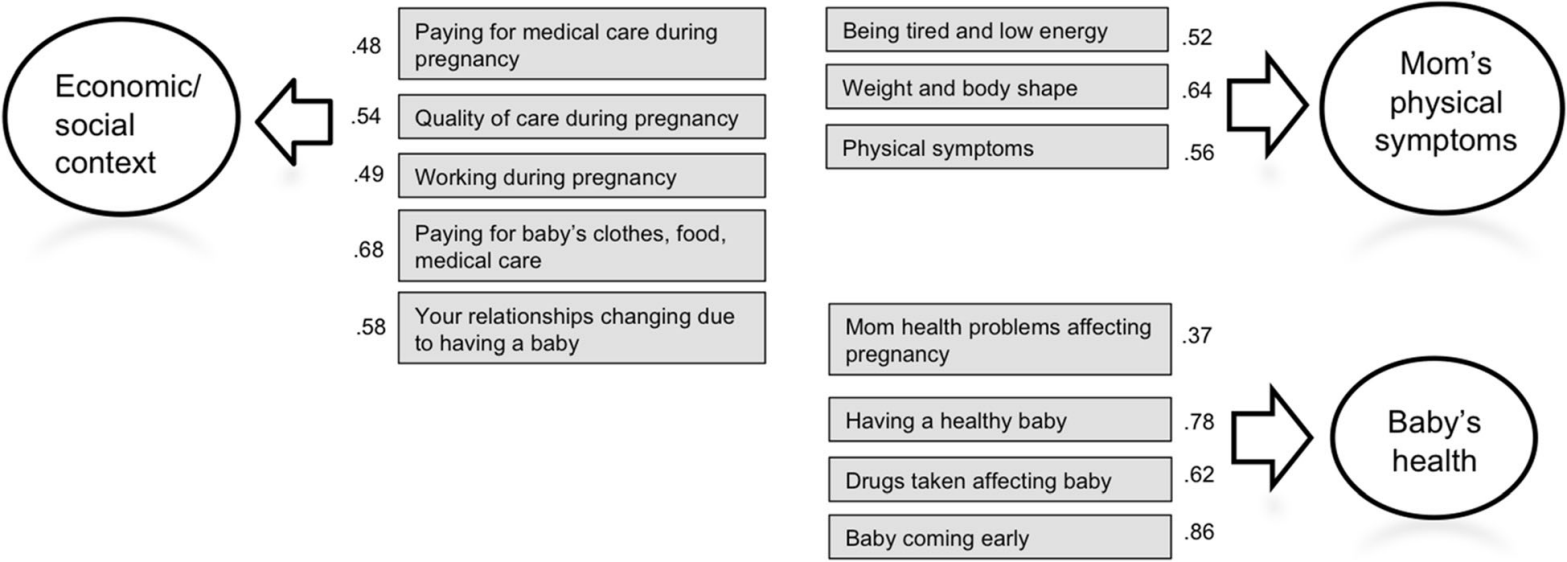
Three Factors of the Prenatal Distress Questionnaire with Factor Loadings

The three factor solution from previous research [38] fit the data adequately (RMSEA = 0.06, CFI = 0.91). While the CFI is slightly below 0.95, the model can still be considered to fit well, considering that there are 12 indicators and three factors [42]. The three factors (Fig. 1) reflect women’s concerns about their physical symptoms during pregnancy, the baby’s health, and their economic situation and relationships. Scores for these factors were then extracted and used in the following regression analyses.

### Pregnancy-Specific Stress and Warmth and Contingent Responsiveness

**Fig. 2:**
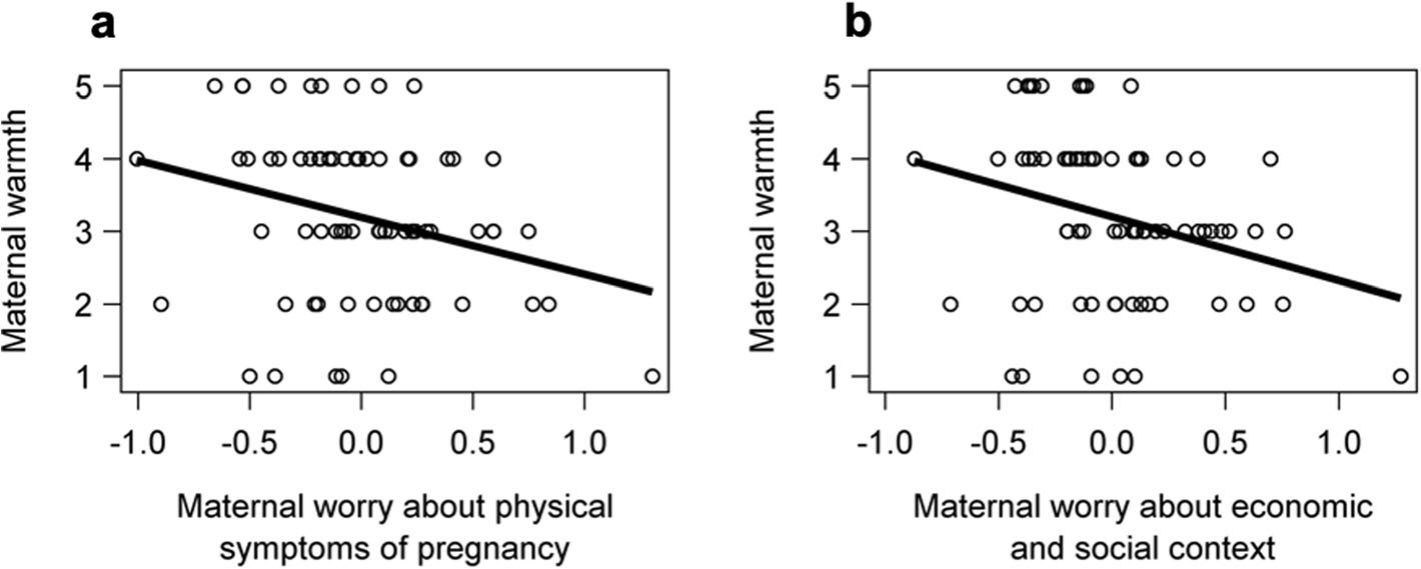
Maternal warmth at 14 months as a function of (**a**) maternal stress about physical symptoms of pregnancy and (**b**) maternal stress about economic/social context during pregnancy

Maternal stress about physical symptoms during pregnancy was significantly and inversely associated with maternal warmth at 14 months (*p* = .01, Table 2; Fig. 2). For a one-unit increase in maternal stress about physical symptoms, the odds of lower warmth versus the other categories combined were 4.17 times greater. Similarly, higher maternal stress about economic/social context during pregnancy was significantly associated with lower maternal warmth at 14 months (*p* = .01; Table 2; Fig. 2). For a one-unit increase in maternal stress about economic/social context, the odds of lower warmth versus the other categories combined were 4.79 times greater. The association between maternal stress about baby health and maternal warmth was marginally significant (*p* = .07). None of the three NuPDQ factors were significantly associated with maternal contingent responsiveness. Maternal warmth and contingent responsiveness were moderately correlated (*r* = .61).

**Table 2:** Maternal pregnancy-specific stress predicts maternal warmth at 14 months

	Observed maternal behavior at 14 months (*n* = 71)			
	Warmth		Contingent responsiveness	
Maternal NuPDQ factor	OR	95 % CI	OR	95 % CI
Physical symptoms	4.17*	1.38, 12.60	1.63	0.56, 4.76
Baby health	1.89	0.95, 3.76	1.61	0.81, 3.19
Economic/social context	4.79*	1.45, 15.90	2.20	0.69, 7.05

Note. Analyses control for baby sex and maternal age. Ordinal values of maternal warmth and contingent responsiveness were as follows: almost never, some of the time, half the time, most of the time, almost always. Statistically significant odd ratios are indicated by an asterisk.

*OR* proportional odds ratio; *CI* confidence interval

In the final model, we did not control for perceived stress at the time of the caregiving observations (the 14-month time point) because of the high correlation between pregnancy-specific stress during mid-gestation and perceived stress at 14 months postpartum (*r* = .49, *p* < .0001 for NuPDQ total score; *r* = .45, *p* < .0001 for stress about physical symptoms; *r* = .42, *p* < .001 for stress about the baby; *r* = .48, *p* < .0001 for stress about the social and economic context).

## Discussion

Previous studies have found that maternal depression or anxiety predict lower caregiving sensitivity [45]. We add to the literature by focusing on pregnancy-specific stress and examining its correlation with caregiving sensitivity in adolescent mothers, most of whom are LatinX ethnic minorities in our sample. We found that greater prenatal stress about physical symptoms of pregnancy and pregnancy-related social and economic concerns were significantly associated with reduced maternal warmth but not contingent responsiveness at 14 months postpartum. Feelings of stress related to pregnancy may make the transition to parenthood more difficult for adolescents and represent some of the earliest modifiable indicators of later risk to the child.

In terms of dimensions of caregiving sensitivity, prenatal stress about physical symptoms and social/economic concerns were significantly related to lower maternal warmth but not maternal contingent responsiveness. Maternal warmth is a stronger reflection of the level of maternal positive affect compared to contingent responsiveness, which largely reflects maternal attunement to child cues and her prompt responses to them [46, 47]. Pregnancy-specific stress may have more of an effect on the affective nature of parenting behavior rather than the parent’s overall level of responsiveness. Maternal warmth, which includes sensitive physical touch and affection, is critical to children’s development of attachment security, emotion regulation, and social orienting [48, 49]. Thus, reducing maternal pregnancy-specific stress may have down-stream positive impacts on cultivating mother-child interactions that support children’s social-emotional development. Prevention and intervention programs that start prenatally are ideally timed to relieve pregnancy-specific stress and in turn set the stage for positive future mother-infant interactions. The prenatal period represents an opportunity to intervene, support, and prepare vulnerable women before they have the added stress of parenting a newborn.

Practical Resources for Effective Postpartum Parenting is an example of an intervention program that aims to treat at-risk women by promoting maternally–mediated behavioral changes in their infants, while also including mother–focused skills (e.g., mindfulness). Results from a randomized control trial indicate that this novel, brief intervention reduced maternal symptoms of anxiety and depression, particularly at 6 weeks postpartum, although symptomology in the sample was sub-clinical [50]. Such interventions can leverage the unique, dyadic nature of the transition to parenting, addressing mothers’ prenatal distress as a way to focus on the health of both mothers and babies.

Integrated interventions to address pregnancy-specific stress in prenatal care could also address socioeconomic concerns like housing, childcare, or access to government benefits [51–56]. Home visiting programs such as the Nurse-Family Partnership start in the prenatal period and assist women with achieving economic stability, prior to also guiding them in providing positive care to their children. Another policy consideration could involve extending the period of Medicaid coverage for postpartum women, given that coverage ends 60 days after birth. Such approaches could be a valuable means of relieving pregnancy-related stress related to the challenges of adolescent childbearing in contexts of socioeconomic disadvantage.

Several limitations of this study should be noted. First, despite its longitudinal design, this study is not equipped to make causal inferences, due in part to its correlational (non-experimental) design. Second, there was attrition from when the study started during pregnancy to the 14-month time point when observer-rated maternal caregiving data were collected. We did not find evidence of differential attrition by prenatal stress or demographic variables, however. Third, although it is possible that the range of maternal sensitivity in adolescent mothers may be shifted lower (compared to older mothers), there was no evidence that a restricted range of contingent responsiveness influenced the results. Fourth, given that our sample consisted of primarily women of color, the pregnancy-specific stress measure may not have captured the full range of types of stressors relevant in pregnancy. Specifically, the NuPDQ does not include questions on racism or discrimination. Finally, these findings in a group of primarily LatinX adolescents, may not be generalizable to other ethnic groups.

## Conclusions

Our results provide a finer-grained picture of prenatal stress and encourage further studies on how pregnancy-specific stress is related to caregiving sensitivity in at-risk mother-infant dyads. Studies building on the Developmental Origins of Adult Health and Disease model that examine the association between prenatal stress and child outcomes should consider caregiving sensitivity as a factor likely involved in these associations.

With respect to clinical implications, these findings also may facilitate earlier identification of mothers who may have later difficulty providing sensitive care to their children, opening a window of opportunity to intervene earlier, during pregnancy, to support healthy development in mother-child dyads. This approach is consistent with the focus of recent U.S. Preventive Services Task Force efforts for prevention of perinatal depression and its effects on children [21].

## Data Availability

The datasets analyzed during the current study are available from the corresponding author on reasonable request.

## Abbreviations

NuPDQ: Revised Prenatal Distress Questionnaire
CFA: Confirmatory factor analysis
RMSEA: Root Mean Square Error of Approximation
CFI: Comparative Fit Index
